# The Effect of Physical Therapy on Somatosensory Tinnitus

**DOI:** 10.3390/jcm13123496

**Published:** 2024-06-14

**Authors:** Hong-Zhe Yu, Jia-Min Gong, Guo-Wei Hong, Ruo-Qiao Zhou, Xin-Ping Fu, Ting Fan, Yu-Qing Zheng, Ying-Qiu Peng, Jian Li, Yun-Feng Wang

**Affiliations:** 1 ENT Institute and Department of Otorhinolaryngology, Eye & ENT Hospital, Fudan University, Shanghai 200031, China; yuhongzhe711@163.com (H.-Z.Y.); gjmin123@163.com (J.-M.G.); hongguowei2023@163.com (G.-W.H.); zhou_rq@126.com (R.-Q.Z.); xinping_fu@163.com (X.-P.F.); 20111260019@fudan.edu.cn (T.F.); pengyingqiu0525@163.com (Y.-Q.P.); 2 NHC Key Laboratory of Hearing Medicine, Fudan University, Shanghai 200031, China; 3 Department of Medical Technology and Information Engineering, Zhejiang Chinese Medical University, Hangzhou 310053, China; qyzl775@163.com; 4 Clinical Laboratory Center, Children’s Hospital of Fudan University, National Children’s Medical Center, Shanghai 201102, China

**Keywords:** somatosensory tinnitus, physical therapy, clinical effects

## Abstract

**Objective**: The objective of this work was to assess the effect of physical therapy in patients with somatosensory tinnitus (ST) and explore the influence of physical therapy on clinical variables obtained before treatment. **Methods**: A total of 43 patients with ST were randomized to the immediate-start group (*n* = 20) and delayed-start group (*n* = 23). All patients received physical therapy for 1 week (seven sessions). Each session lasted 60 min. The Visual Analogue Scale (VAS), Tinnitus Handicap Inventory (THI), and numerical pain rating scale (NPRS) scores were documented at baseline and after treatment (week 1) for all patients. For subjects in the immediate-start group, the THI, VAS, and NPRS scores were measured after therapy (weeks 6, 9, and 12, respectively). Medical history characteristic functional activity scale (HCFA) scores were measured at baseline to assess the association between somatic symptoms and tinnitus. **Results**: At week 1, VAS, THI, and NPRS scores of patients in the immediate-start group were improved by 1.25 ± 1.59, 11.10 ± 15.10, and 0.95 ± 1.54 points, respectively, and were significantly higher than those in the delayed-start group (*p* < 0.05). The change in VAS, THI, and NPRS scores in the treatment group was significantly positively correlated with the scores of the HCFA before treatment (r = 0.786, *p* < 0.001; r = 0.680, *p* = 0.001; r = 0.796, *p* < 0.001). There was no significant difference in THI, VAS, and NPRS scores among patients in the immediate-start group between weeks 1, 6, 9, and 12 after treatment (*p* > 0.05). **Conclusions**: Although more participants were necessary in the further study, the study implies that physical therapy can reduce physical pain, improve tinnitus symptoms, and quality of life in ST patients without hearing loss, and the short-term curative effect is stable, especially for tinnitus patients with clear somatic symptoms.

## 1. Introduction

Tinnitus is the perception of sound in the absence of external sound stimulation [1] and has a variety of causes. Table 1 shows the summary of the etiology and probable pathogenesis of tinnitus. It is considered a symptom, not a disease itself [2]. More than 10% (11.9–30.3%) of the adult population suffer from tinnitus [3], and the prevalence of troublesome tinnitus increases with age [1]. The treatment of tinnitus has always been a thorny global issue, and tinnitus seriously affects the quality of life. Somatosensory tinnitus is a well-recognized subtype of tinnitus associated with activating the somatosensory, somatomotor, and visuomotor systems [4]. A key feature of somatosensory tinnitus is that it is evoked or modulated by physical contact or movement, such as forceful muscle contractions of the temporomandibular joint (TMJ), head and neck, and limbs. This means that the psychoacoustic properties of tinnitus (loudness and pitch) may change instantaneously in response to different stimuli.

Different countries and institutions have reported different prevalence rates. Based on the characteristics of somatic tinnitus, its prevalence depends largely on whether the tinnitus is induced by self-reported or expert-guided actions. Self-reporting may underestimate patients’ conditions [17]; for example, the prevalence of somatosensory tinnitus is higher when patients are specifically asked rather than self-report [18]. The characteristics of somatosensory tinnitus were recently investigated in a large cohort in the United Kingdom, and the authors found a prevalence of 16% for a self-reported ability to modulate tinnitus [17]. However, Sanchez et al., (2002) [18] reported that 65.3% of 121 patients developed tinnitus after performing a series of body movements. Previous studies have shown that patients with cervicogenic somatic tinnitus account for 36~43% of the entire tinnitus population [19,20,21].

Although somatosensory tinnitus is common, its pathogenesis remains poorly understood. Studies have reported a link between proprioceptive and nociceptive afferents of the cochlear nuclei and neck—this could explain the ipsilateral correlation with tinnitus in the context of muscle tension [22,23]. The cochlear nucleus plays a role in receiving information from the cochlear hair cells and is the first central nucleus of the auditory pathway. The relationship between auditory and non-auditory pathways has been reported, which might be because many neurons of the extralemniscal system receive information from other sensorial tracts, such as the somatosensory system [24]. Many studies have suggested that this type of tinnitus is closely related to abnormal cross-modal plasticity of somatosensory and auditory interactions and that the resulting somatic modulations of tinnitus arise from abnormal auditory neural interactions—distortion of normal synaptic activity within the central nervous system [25]. Sanchez et al., (2007) [26] reported on “the information triggered by muscle contraction carried by the somatosensory system, and, upon reaching the cuneiform nucleus, may influence through its projection over the auditory pathway due to an overactivity in the cochlear nucleus.” Particularly, modulation of dorsal cochlear neuronal hyperactivity is triggered by stimulation of specific ipsilateral cranial nerves (i.e., branches of the trigeminal nerve), which explains how ipsilateral tinnitus can be modulated by manipulations of the head and neck [27]. That is, the intensity disturbance between the trigeminal nerve and inner ear neural pathways in the brainstem causes the patient to experience a stress signal that is misinterpreted as sound, producing somatosensory tinnitus.

Several treatments for somatosensory tinnitus have been proposed, such as physical therapy [28] (including temporomandibular disorder (TMD) treatment, chiropractic, muscle relaxation, etc.), electrical stimulation therapy [29], and pharmaceutical treatment [30]. According to existing literature reports, physical therapy, such as stabilization splint in the presence of TMJ disease, and osteopathic manipulation of the cervical spine in the presence of head and neck muscle disease, are currently more effective methods [4]. However, there is no high-quality evidence for the effect of physical therapy treatment in tinnitus patients, mainly limited by the need for (1) clearer participant inclusion criteria and (2) larger sample experiments to provide more evidence for physiotherapy [28]. Integrating multidisciplinary strategies and formulating high-efficiency strategies according to each patient’s tinnitus characteristics requires further research. The aim of this study is to explore the prognosis analysis of physical therapy and its curative effect on somatosensory tinnitus.

## 2. Materials and Methods

### 2.1. Study Design

Consecutive patients with somatosensory tinnitus were recruited from the Department of Otorhinolaryngology of Eye and ENT Hospital of Fudan University between February 2019 and May 2019. Participants over 18 years of age who met the diagnostic criteria for somatosensory tinnitus [31,32,33] were included in the study. Specifically, patients were included if they had symptoms of tinnitus which had been stable for at least three months and at least one of the following additional features: (1) tinnitus accompanied by recurrent, intermittent attacks of pain in the head, neck, shoulder, back, and/or upper limb regions; (2) changes in head and neck posture leading to the aggravation or induction of tinnitus; or (3) aggravation of muscle pain or spasm in the head, neck, shoulder, back, and/or upper limb regions synchronized with the occurrence/exacerbation of tinnitus. It was ensured that included patients had not received any treatment for the somatosensory tinnitus. The exclusion criteria were: (1) previous physical therapy for tinnitus in the past 5 years; (2) a history of ear trauma; (3) previous ear surgery; (4) a history of sensorineural deafness; (5) a history of psychiatric disorders; and (6) a history of neural and central auditory diseases. This study was approved by the ethics committee of the hospital (Ref. 2022137), and the Clinical Trials Registry No. is ChiCTR1900020746. All participants provided informed written consent for inclusion in the study.

Patients were randomized into the immediate-start group or into the delayed-start group in a 1:1 ratio, ensuring an even distribution based on sex and age. Concealed allocation was conducted by an external researcher not involved in participant recruitment using a computer-generated randomized list. The assessor performing the clinical tests was blinded to the allocation of the patients in the immediate-start and delayed-start groups. The immediate-start group patients received treatment immediately after the diagnosis of somatosensory tinnitus, while patients in the delayed-start group were placed on a waiting list for 1 week. All the patients were treated with a 1-week program targeting the head and neck, shoulders, and upper limbs, which was a supervised physical therapy program [34,35,36].

### 2.2. Intervention

A systematic literature review of six studies involving physical therapy (cervical spine treatment and TMJ treatment) showed a positive effect on the severity of tinnitus [34]. In this study, physical therapy was administered once a day for a consecutive week by a senior otolaryngologist with a background in sports rehabilitation and an audiologist trained in physical therapy, with each treatment lasting for about 60 min. The treatments used in this study included the following three parts: head and neck muscle strength training, cervical muscle stretch training, and cervical joint activity training. Head and neck muscle strength training was carried out by a clinician. The clinician crossed their hands and in turn exerted pressure on the patient’s forehead, behind the occipital bone, and on the left and right sides of the cheek, while the patient attempted to perform forward flexion, backward extension, and left and right lateral flexion of the neck against resistance. Patients could also exercise with their own hands under the guidance of a clinician. Cervical muscle stretching exercises: The clinician instructed patients to stretch themselves. Neck stretch: The patient lifted the chest, slightly extend the head and neck, sunk the shoulders, retreated the lower forehead, and felt a slight stretch below the occipital bone. Neck rotation: The patient gently relaxed the muscles after rotating the head and neck. Muscle relaxation through stretching exercises can modulate tinnitus, which may play an important role in the treatment of tinnitus [25]. Cervical joint activity training: The participant began the exercise with the cervical spine in a neutral position (straight neck) with the head facing forward and the mandible adducted to a 0-degree angle. (1) The patient’s neck flexion was 35 degrees forward, involving the longus neck muscles; longus capitis muscles; and anterior, middle, and posterior scalene muscles. The patient’s neck flexion was 35 degrees backward, involving the sternocleidomastoid muscles, splenius cervicis, longissimus dorsi muscles, and sacral spinalis muscles. (2) The patient’s neck left and right lateral flexion was adjusted to 45 degrees, involving the anterior, middle, and posterior scalene muscles. (3) The patient’s neck was rotated left and right to 30 degrees, involving the splenius cervicis muscles. This physical therapy followed the regimen described by Maitland et al., (2005) [37]. Since the treatments were administered one-to-one by a clinician, it was ensured that all patients were fully compliant with the therapy.

### 2.3. Medical History and Tinnitus Characteristics

The age, gender, side of tinnitus, and specific questions related to somatosensory tinnitus were inventoried at baseline. These specific questions are related to the diagnostic criteria for ST [32].

When recording medical history at baseline, specific characteristics of somatosensory tinnitus were recorded, summarized, and divided into four items, each comprising 10% of the weight, with a maximum score of 40 points. It was named the History and Characteristic Functional Activity Scale (HCFA) in this study. The specific four items are as follows:

(1) Definite head and neck trauma or history of cervical vertebra;
(2) Inappropriate changes in head and neck posture during rest and activity inducing or aggravating tinnitus;
(3) Forcibly opening the mouth or biting teeth or severe bruxism at night leading to tinnitus changes;
(4) Repeated intermittent shoulder and back pain of upper limbs accompanied by tinnitus changes.

### 2.4. Outcome Measures

To measure the severity of tinnitus, we considered using the validated Mandarin version of the Tinnitus Handicap Inventory (THI) [38] and Visual Analogue Scale (VAS) [39] as the main outcomes.

The THI is a self-report questionnaire and consists of 25 items on three scales, functional (11 items), catastrophic (5 items), and emotional (9 items), that assess the influence of tinnitus in daily life [40]. There are three possible responses to each item: yes = 4 points, sometimes = 2 points, or no = 0 points. The total score range is from 0 to 100 points, with a higher score indicating a greater perceived severity level of tinnitus. The internal reliability (Cronbach’s α, 0.93) and test–retest reliability (Pearson correlation, 0.98) of the THI are excellent [38].

The subjective loudness and annoyance of tinnitus in daily life were rated using the VAS. The patients were asked to indicate their tinnitus effects in their daily life by selecting a mark along a line ranging from zero to ten. Zero indicated that patients did not have audible tinnitus, were not annoyed, and their life was not affected by tinnitus at all, while ten indicated that the patients had extremely loud tinnitus, were considerably annoyed, and their life was affected by tinnitus all the time [39].

Patients included in this study may also have experienced somatic spasm pain. The numerical pain rating scale (NPRS: 0 = no pain, 10 = maximum pain) was used to assess the intensity of somatic spasm pain [41].

These three questionnaires were administered at baseline and after treatment (week 1) for all patients. For subjects in the immediate-start group, the follow-up assessment (THI, VAS, and NPRS) was conducted after therapy (weeks 6, 9, and 12, respectively).

### 2.5. Statistical Analysis

First, the normality of data was investigated using the Shapiro–Wilk test. Baseline comparability (*p* > 0.05) of both groups was analyzed using descriptive statistics, independent samples *t*-tests, and chi-square tests.

Second, the change in the VAS (∆VAS), THI (∆THI), and NPRS (∆NPRS) scores from baseline to week 1 was calculated. With these three new variables, we were able to compare the evolution in the VAS, THI, and NPRS scores between both groups to investigate the effect of physical therapy. The differences between both groups were compared using independent samples *t*-tests for normally distributed data, and Mann–Whitney U-tests for non-normally distributed data.

In the third step of the analysis, correlations between the baseline of age, pure tone average (PTA), VAS, THI, NPRS, and HCFA and the ∆VAS, ∆THI, and ∆NPRS scores were investigated using Pearson or Spearman correlation coefficients depending on the normality of the data. The *t*-tests were used to determine the relationship between dichotomous variables (sex, duration of tinnitus, the pitch of tinnitus) and the ∆VAS, ∆THI, and ∆NPRS scores, and the chi-square test was adopted to investigate the relationship between unordered multinomial variables (the side of tinnitus) and the ∆VAS, ∆THI, and ∆NPRS scores in the treatment group.

Then, a univariate regression analysis was performed and was used to predict the effect of physical therapy. Potential prognostic indicators that correlated significantly (*p* < 0.10) with the evolution in outcome measures from baseline to week 1 were included in the regression analysis. Significance levels were chosen to allow a broad screening for potential prognostic indicators [42].

Additionally, differences in outcome measures between baseline, post-treatment, and follow-up were calculated using the paired samples *t*-test to investigate the effect stability of physical therapy in this study.

All statistical analyses were conducted with IBM SPSS Statistics, v28 (IBM Corp., Armonk, NY, USA), with *p* < 0.05 as the significance level.

## 3. Results

### 3.1. Patients and Tinnitus Characteristics

Forty-three patients were randomly assigned to the immediate-start group or delayed-start group. The mean age of patients in the immediate-start group was 27.95 years (range: 19–43 years), and that of subjects in the delayed-start group was 29.96 years (range: 19–45 years). The duration of tinnitus was 7.05 ± 2.13 in the immediate-start group and 7.18 ± 1.96 in the delayed-start group, with no statistically significant difference. Tinnitus was unilateral in 16 patients (80%) in the immediate-start group and 21 patients (91.3%) in the delayed-start group. The average PTA thresholds were 20.55 ± 3.46 dB HL for the immediate-start group and 20.17 ± 5.36 dB HL for the delayed-start group, and no significant differences were found. Demographic measures for both groups are presented in Table 2. No significant differences were found at baseline between the immediate-start and delayed-start groups (Table 2). Three participants in the immediate-start group were not contacted after being assigned.

### 3.2. The Effect of Physical Therapy

**Figure 1:**
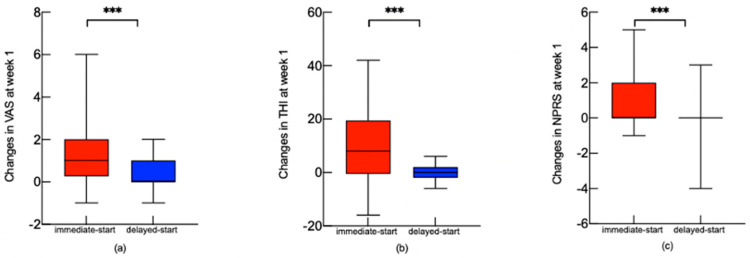
The effect of physical therapy. The alteration in the VAS, THI, NPRS scores after the 1-week treatment period in immediate−start and delayed−start groups. The change in VAS, THI, and NPRS scores after the 1-week treatment period in immediate−start and delayed−start groups is shown by the red box and blue box, respectively. (**a**) Changes in VAS at week 1 in both groups. (**b**) Changes in THI at week 1 in both groups. (**c**) Changes in NPRS at week 1 in both groups. Independent samples *t*-tests were used, and the results showed the significant difference in the evolution in VAS, THI, and NPRS scores at week 1 between the immediate−start and delayed−start groups. VAS: Visual Analogue Scale; THI: Tinnitus Handicap Inventory; NPRS: Numerical Pain Rating Scale. *** *p* < 0.001.

After a 1-week treatment period, the scores of the VAS, THI, and NPRS for the immediate-start group were 2.40 ± 1.00, 28.50 ± 14.62, and 2.55 ± 0.89, respectively, significantly different from the baseline (*p* < 0.05). ∆VAS scores for the immediate-start and delayed-start groups were 1.25 ± 1.59 and 0.13 ± 0.82, respectively, showing significant differences (*p* = 0.005). The ∆THI score for the immediate-start group was 11.10 ± 15.10, which is significantly higher than that of the delayed-start group (*p* = 0.004). Immediately after treatment, the average NPRS score for the immediate-start group decreased statistically significantly by 0.95 ± 1.54 compared with the delayed-start group (*p* = 0.025). Figure 1 shows the significant difference in evolution between the immediate-start and delayed-start groups. In our study, 45% of the patients (*n* = 20) had decreased VAS, THI, and NPRS scores in the immediate-start group after a 1-week treatment period.

### 3.3. The Relationship between the Baseline and the Effect Enhancement of Physical Therapy for the Immediate-Start Group

**Figure 2:**
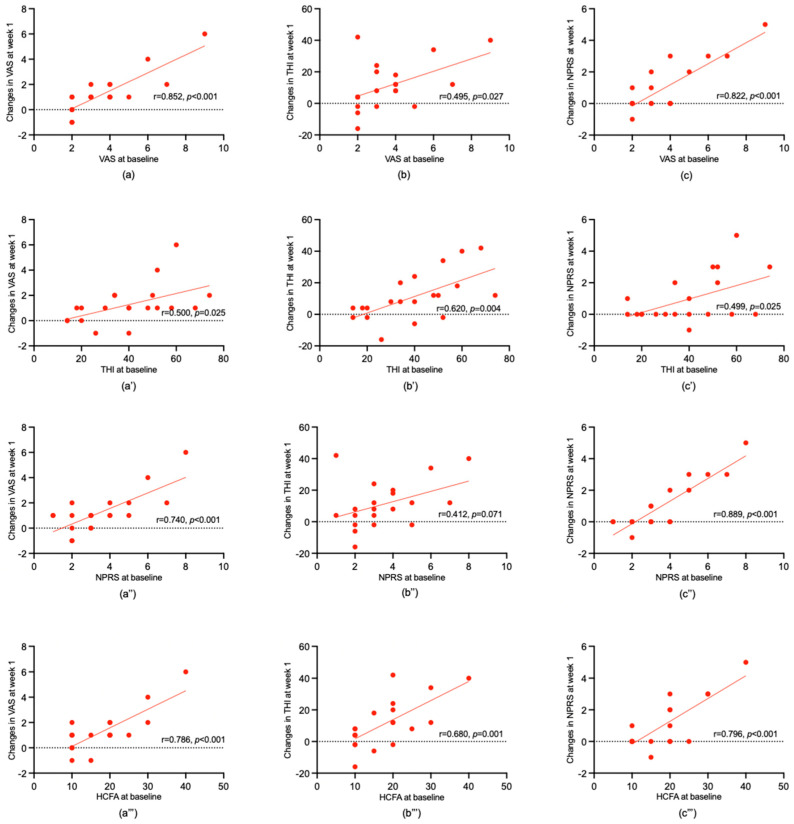
Correlation of the baseline of VAS, THI, NPRS, and HCFA scores with the effect of physical therapy in the immediate−start group. Pearson correlations were used, and the results showed the significant relationship between the baseline of VAS, THI, NPRS, and HCFA scores and ∆ VAS (**a**,**a′**,**a″**,**a‴**), THI (**b**,**b′**,**b″**,**b‴**), and NPRS (**c**,**c′**,**c″**,**c‴**). VAS: Visual Analogue Scale; THI: Tinnitus Handicap Inventory; NPRS: Numerical Pain Rating Scale; HCFA: History and Characteristic Functional Activity Scale.

The *t*-tests were used to analyze the association between sex, duration of tinnitus, the pitch of tinnitus and ∆VAS, ∆THI, and ∆NPRS scores. The results revealed that there were no significant ∆VAS, ∆THI, or ∆NPRS scores associated with sex, duration of tinnitus, or the pitch of the tinnitus. The chi-square test did not show any significant correlations between the side of tinnitus and ∆VAS, ∆THI, and ∆NPRS scores. Figure 2 shows a significant positive relationship between THI, VAS, and NPRS scores at baseline and ∆VAS, ∆THI, and ∆NPRS scores (all *p* < 0.05, except for the relationship between NPRS scores at baseline and ∆THI). It is noted that the HCFA scores at baseline were significantly positively related to ∆VAS, ∆THI, and ∆NPRS scores (r = 0.786, *p* < 0.001; r = 0.680, *p* = 0.001; r = 0.796, *p* < 0.001, respectively). 

Table 3 shows the prognostic indicators for a clinically relevant reduction in VAS, THI, and NPRS scores after the 1-week treatment period. Taken together, these results suggest that the severity of tinnitus before treatment and the somatic modulation at the baseline were positively correlated to the degree of improvement for tinnitus after 1 week of physical therapy.

### 3.4. The Comparison of the THI, VAS, and NPRS Scores after 1, 6, 9, and 12 Weeks of Follow-Up in the Immediate-Start Group

**Figure 3:**
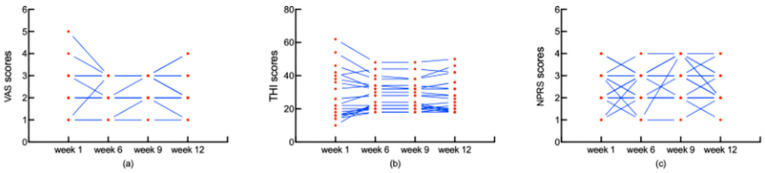
The comparison of the VAS (**a**), THI (**b**), and NPRS (**c**) scores after 1, 6, 9, and 12 weeks of follow-up in the immediate-start group. The red dot means the scores of VAS (**a**), THI (**b**), and NPRS (**c**) at week 1, 6, 9 and 12, respectively. The blue lines mean the change for scores of VAS (**a**), THI (**b**), and NPRS (**c**) at week 1, 6, 9 and 12 for the patients. The paired samples *t*-test shows that there was no significant difference in THI, VAS, and NPRS scores after 1, 6, 9, and 12 weeks. VAS: Visual Analogue Scale; THI: Tinnitus Handicap Inventory; NPRS: Numerical Pain Rating Scale.

There were no significant differences in the THI, VAS, and NPRS scores between the follow-up data (after 1, 6, 9, and 12 weeks of treatment) (Figure 3). This effect was maintained after the 12-week follow-up period in 35% of the patients (7 out of 20).

## 4. Discussion

The present study was designed to determine the effect of physical therapy on somatic tinnitus and prognostic analysis. The results of this study show that somatic tinnitus is more common in younger patients, which is consistent with the study conducted by Ward et al. [17]. Regarding the gender distribution for somatic tinnitus, our results showed that there were nearly 1.3 to 1.5 times more male patients than females, which corroborates the findings of other studies [4,17]. However, there were some researchers who reported the reverse direction, that the female gender was more strongly related to somatic modulation [43,44]. Our study found that the numbers of patients with unilateral somatic tinnitus was higher than the number of bilateral somatic tinnitus patients. In agreement with the present results, previous studies have demonstrated a higher prevalence of unilateral somatic tinnitus than bilateral tinnitus related to somatic modulation [4,43]. Another finding in our study is that there were 6.6 times more somatic tinnitus patients with high pitch than low-pitch somatic tinnitus patients, which also accords with another study [4]. However, this outcome is contrary to previous studies, which have documented that the number of patients with high pitch was lower than the number of patients with low pitch [43].

Regarding the effect of physical therapy, the scores of three questionnaires were used to assess the tinnitus severity and the pain of the somatic spasm. The scores of the VAS, THI, and NPRS for the immediate-start group after the 1-week treatment period significantly improved compared with the previous treatment. The change in VAS, THI, and NPRS scores for the immediate-start group at week 1 compared to the delayed-start group was statistically significant. Especially, the change in THI at week 1 for the immediate-start group was 11.10 ± 15.10, which is similar to the 15-point reduction in another study [45] suggested to be clinically significant. However, the decrease in the Tinnitus Functional Index (TFI) was 11.9 ± 21.4 in the similar study [34], and the standard deviations were larger than in our study. This might be owing to the two different questionnaires used to measure the severity of tinnitus. A study [46] also proposed similar concerns regarding the insufficient responsiveness of the TFI for the alteration in somatic tinnitus. In our results, the scores of the VAS, THI, and NPRS for 45% of patients (*n* = 20) were reduced. This finding supports evidence from clinical observations [34,45] that nearly half of the subjects reported the improvement in somatic tinnitus after the physical treatment. These results suggest that the physical therapy was effective for patients with somatic tinnitus, which was also reported by other studies [34,44,45,47]. The current study found that the scores of the VAS, THI, and NPRS at week 1 were not significantly different from follow-up data (weeks 6, 9, 12), and the effect of physical therapy was maintained at week 12 in 35% of the patients (7 out of 20), indicating that physical therapy has a stable effect on somatosensory tinnitus. This finding was also reported by another study [34]. The subjects exhibited significant therapeutic effects in this study where physical therapy mainly focused on the head and neck, suggesting that the connection between the somatosensory and auditory pathways in somatosensory tinnitus patients is more abundant at the head and neck level, and physical stimulation in the head and neck can improve the symptoms of somatosensory tinnitus. Also, there is evidence of existing neural connections between somatosensory and auditory systems, and somatic tinnitus may be modulated through non-audiology stimuli [31].

Another important finding is the significant association between the higher scores of the VAS, THI, NPRS, and HCFA before treatment and the effect of physical therapy, which revealed that severe tinnitus and obvious somatic characteristics at baseline are significantly related to physical therapy improvement. These findings may be explained by the higher scores of the VAS, THI, NPRS, and HCFA, which may correspond to more complaints of tinnitus and the somatosensory system more significantly influencing the tinnitus. Since the physical therapy in this study aims to ameliorate the severity of tinnitus and the somatic pain to the greatest extent, a larger improvement can be expected in patients with more complaints before treatment. This finding is in agreement with another study [34], which showed that tinnitus co-varied with cervical postures is a prognostic indicator for the reduction in the TFI after treatment. Furthermore, this finding broadly supports the work of other studies in this area, linking the higher scores of tinnitus severity at baseline with the more significant improvement after physical therapy [44,47]. A similar study [34] reported that low-pitch tinnitus could be a prognostic indicator of a positive outcome after therapy. It has been suggested that the short duration of tinnitus and younger women were also the prognostic indexes for the treatment [44], whereas our results have not shown other variables statistically significantly related to the effect of physical therapy. A possible explanation for this is that, in the participants included in our study, the male population was larger, and the tinnitus tone was mainly high-frequency. For future studies, it is necessary to collect more precise baseline data and include more subjects to explore the tinnitus characteristics at baseline and influence the outcome of physical therapy. Furthermore, the follow-up data for the delayed-start group should be collected to further analyze the stability of physical therapy for ST patients. This could contribute to more patients with somatosensory tinnitus benefiting from physical therapy.

## 5. Conclusions

This study further demonstrated that physical therapy could provide greater health benefits for ST patients without hearing loss, especially for severe tinnitus and obvious somatosensory characteristics. Further studies with a larger sample size using more detailed baseline data are needed to predict the prognostic effects of physical therapy.

## Figures and Tables

**Table 1:** Summary of etiology and probable pathogenesis of tinnitus.

Etiology of Tinnitus	Probable Pathogenesis
Vascular disorders (arterial bruits, arteriovenous shunts, paraganglioma, venous hums)	Tinnitus caused by vascular disorders (arterial bruits, arteriovenous shunts, paraganglioma, venous hums) is usually associated with blood flow or vascular abnormalities in the head or neck near the cochlea. When the blood vessels in the skull or neck are abnormal, they may stimulate hair cells in the cochlea (part of the inner ear) and transmit to the cochlea through the bony structure, so that tinnitus consistent with the heartbeat can be heard, causing pulsatile tinnitus [5].
Neurologic disorders	Tinnitus may be related to abnormalities in the central nervous system and problems in the limbic and autonomic nervous systems. The limbic system belongs to the non-auditory central part, mainly composed of the hippocampal formation, parahippocampal gyrus, cingulate, and amygdala. Some studies have shown that the limbic system is involved in the formation of tinnitus. Nervous system disorders (such as anxiety, depression) are closely related to parts of the limbic system such as the hippocampus and prefrontal lobe, resulting in tinnitus [6].
Eustachian tube dysfunction	The tension of the peripheral muscles of the eustachian tube produces asynchronous pulsatile tinnitus, while the incomplete closure of the eustachian tube reduces the effect of body sound masking, and the internal noise is abnormally transmitted to the ear, resulting in the self-sound of breathing and the perception of unusually loud sound, which makes it easier for patients to hear body voices [7].
Other somatic disorders (temporomandibular joint (TMJ) dysfunction)	Hypertonia of the masticatory muscles due to TMD and excessive tension of ligaments close to the ossicles of the middle ear may be associated with somatosensory tinnitus, and these abnormal signals may affect the auditory pathway through the cochlear nerve. However, somatosensory tinnitus persists after the auditory nerve is severed, so these peripheral system explanations cannot be the sole explanation for the source of somatosensory tinnitus. Furthermore, the trigeminal nucleus in the somatosensory system receives sensory inputs from the temporomandibular joint (TMJ) and projects them to the cochlear nucleus (CN) through different nerve fibers, which allows the somatosensory system to affect the auditory system by altering the spontaneous rate and the synchronized firing patterns among neurons in the hypothalamus or auditory cortex, resulting in tinnitus [8,9].
Originating from auditory system:	
(1) ototoxic medications	Drug-induced deafness is mainly due to the imbalance of the ion concentration in the endolymph as a result of the toxicity of drugs, which damages hair cells, spiral ganglia, and vascular veins in cochlea, leading to tinnitus [10].
(2) presbycusis	With the increase in age, the inhibitory function of the auditory center on auditory afferent information is gradually weakened, which leads to further changes in the auditory system and produces tinnitus [11].
(3) otosclerosis	Conductive hearing loss, the loss of cochlear hair cells in the inner ear, and abnormalities in peripheral sensory pathways all lead to hearing deprivation. This deprivation activates neuroplasticity, ultimately resulting in the production of tinnitus. Furthermore, dysfunction of the middle ear muscles can result in alterations in the stimulation of the somatosensory system, ultimately leading to the development of tinnitus [12,13].
(4) vestibular schwannoma	The ephaptic coupling of nerve fibers through microvascular compression or other pathways can lead to the synchronous firing of many nerve cells, which may be associated with the development of tinnitus. Furthermore, aberrant vestibulocochlear nerve input signals and nerve damage can lead to auditory deprivation and trigger neuroplasticity, resulting in the development of tinnitus [14,15].
(5) Chiari malformations	Elevated intracranial pressure caused by hydrocephalus and obstructed cerebrospinal fluid outflow can lead to pulsatile tinnitus. Of course, it is not ruled out that the pressure of the cerebellar tonsil on the vestibulocochlear nerve causes non-pulsating tinnitus [12,15].
(6) noise-induced hearing loss	Noise deafness is mainly caused by noise changing the transmission of neurotransmitters in the auditory central nervous system, resulting in abnormal excitation of the auditory cortex to produce tinnitus [16].

**Table 2:** Patients’ characteristics at baseline.

Characteristic	Total (*n* = 43)	Immediate-Start Group (*n* = 20)	Delayed-Start Group (*n* = 23)	*p* Value
Age (years)	29.02 ± 6.73 (19,45)	27.95 ± 6.10 (19,43)	29.96 ± 7.23 (19,45)	0.335
Sex				0.820
Male	25 (64.1%)	12 (60%)	13 (56.5%)	
Female	18 (35.9%)	8 (40%)	10 (43.5%)	
Duration of tinnitus (months)	7.10 ± 2.08 (4,10)	7.05 ± 2.13 (4,11)	7.18 ± 1.96 (4,10)	0.168
The side of tinnitus				0.126
Both ears	6 (14.0%)	4 (20%)	2 (8.7%)	
Left ear	13 (30.2%)	4 (20%)	9 (39.1%)	
Right ear	24 (55.8%)	12 (60%)	12 (52.2%)	
Tinnitus pitch				0.759
High pitch	38 (88.4%)	18 (90%)	20 (87.0%)	
Low pitch	5 (11.6%)	2 (10%)	3 (13.0%)	
PTA (dB HL)	20.35 ± 4.52 (8,25)	20.55 ± 3.46 (13,25)	20.17 ± 5.36 (8,25)	0.789
VAS (scores)	3.95 ± 1.93 (1,9)	3.65 ± 1.90 (2,9)	4.22 ± 1.95 (1,8)	0.342
THI (scores)	40.93 ± 16.70 (14,80)	39.60 ± 17.94 (14,74)	42.09 ± 16.25 (14,80)	0.636
NPRS (scores)	3.84 ± 2.00 (1,8)	3.50 ± 1.91 (1,8)	4.13 ± 2.07 (1,8)	0.308
HCFA (scores)	19.42 ± 8.18 (10,40)	17.75 ± 8.50 (10,40)	20.87 ± 7.78 (10,35)	0.216

For numeric variables, measures are shown as mean ± SD followed by ranges in square brackets. PTA: pure tone average; VAS: Visual Analogue Scale; THI: Tinnitus Handicap Inventory; NPRS: Numerical Pain Rating Scale; HCFA: History and Characteristic Functional Activity Scale.

**Table 3:** The prognostic indicators of clinically relevant improvement in VAS, THI, and NPRS scores after the 1-week treatment period.

Variable (at Baseline)	Univariate Regression Analysis								
	∆VAS				∆THI		∆NPRS		
	F	R^2^	*p*	F	R^2^	*p*	F	R^2^	*p*
VAS	47.704	0.711	<0.001	5.840	0.203	0.027	37.608	0.658	<0.001
THI	5.987	0.208	<0.001	11.255	0.351	0.004	5.965	0.207	0.025
NPRS	21.842	0.523	<0.001	3.670	0.123	0.071	67.718	0.778	<0.001
HCFA	29.051	0.596	<0.001	15.506	0.433	0.001	31.084	0.613	<0.001

VAS: Visual Analogue Scale; THI: Tinnitus Handicap Inventory; NPRS: Numerical Pain Rating Scale; HCFA: History and Characteristic Functional Activity Scale.

## Data Availability

The raw data supporting the conclusions of this article will be made available by the authors on request.
